# Increased lymphangiogenesis in joints of mice with inflammatory arthritis

**DOI:** 10.1186/ar2326

**Published:** 2007-11-12

**Authors:** Qian Zhang, Yan Lu, Steven T Proulx, Ruolin Guo, Zhenqiang Yao, Edward M Schwarz, Brendan F Boyce, Lianping Xing

**Affiliations:** 1 Department of Pathology and Laboratory Medicine. University of Rochester Medical Center, 601 Elmwood Avenue, Rochester, NY 14642, USA; 2 Center for Musculoskeletal Research, University of Rochester Medical Center, 601 Elmwood Avenue, Rochester, NY 14642, USA

## Abstract

Angiogenesis is involved in the pathogenesis of inflammatory arthritis, but little is known about the role of lymphangiogenesis in this setting. Here, we examined whether tumor necrosis factor (TNF) stimulates osteoclast precursors (OCPs) to produce the lymphatic growth factor, vascular endothelial growth factor-C (VEGF-C), and induce lymphangiogenesis. We used TNF-transgenic (Tg) mice and mice with serum-induced arthritis. OCPs were purified by fluorescence-activated cell sorting of CD11b^+^/Gr-1^-/lo ^blood or bone marrow cells and subjected to microarray analysis or were generated from spleen or joint cells and treated with TNF. Expression of VEGFs was analyzed and examined by real-time reverse transcription-polymerase chain reaction and Western blotting. Immunostaining and magnetic resonance imaging were used to quantify lymphatic vessels and volumes of synovium and draining lymph nodes. TNF stimulated VEGF-C expression by OCPs and increased nuclear factor-kappa B (NF-κB) binding to an NF-κB sequence in the *VEGF-C *promoter. OCPs from joints of TNF-Tg mice express high levels of VEGF-C. Lymphatic vessel numbers and size were markedly increased in joint sections of TNF-Tg mice and mice with serum-induced arthritis. The severity of synovitis correlated with draining lymph node size. In summary, TNF induces OCPs to produce VEGF-C through NF-κB, leading to significantly increased lymphangiogenesis in joints of arthritic mice. The lymphatic system may play an important role in the pathogenesis of inflammatory arthritis.

## Introduction

Joint disease in rheumatoid arthritis (RA) is characterized by inflamed hyperplastic synovial tissue or 'pannus' formation [1]. Pannus is composed of various cell types that produce a vast array of inflammatory mediators, including cytokines and chemokines that destroy the extracellular matrix in the joint by direct and indirect mechanisms. Pannus is extremely vascular, providing portals of entry for effector cells to enter the joint from the circulation and mediate joint destruction via autocrine and paracrine mechanisms. As a result of neovascularization, inflammatory cell infiltration, and concomitant synovial cell hyperplasia, the volumes of the synovium and synovial fluid increase, resulting in joint swelling and pain [2]. Thus, inhibition of new blood vessel formation has been proposed as an important therapeutic approach for patients with inflammatory-erosive arthritis [3].

The lymphatic circulation has been known for many years to be an important secondary vascular system to remove fluid, macromolecules, and cells from the interstitial spaces, and it functions as a 'compensatory' system for blood circulation. However, studies of the lymphatic system have been hampered until recently by the lack of markers that definitively distinguish blood from lymphatic vessels and a paucity of knowledge about growth factors specific to lymphatic endothelial cells. Gene array analysis comparing lymphatic endothelial cells and blood vascular endothelial cells has recently identified numerous previously unknown lineage-specific markers for blood and lymphatic vascular endothelium. Newly identified lymphatic endothelium-specific markers include [4] lymphatic endothelial hyaluronan receptor 1 (LYVE-1), prospero-related homeobox 1, vascular endothelial growth factor receptor 3 (VEGFR-3), and the mucin-type transmembrane glycoprotein, podoplanin [5-8].

In studies using these lymphatic markers, several factors, such as VEGF-A, platelet-derived growth factor (PDGF)-BB, and fibroblast growth factor, have been shown to affect lymphangiogenesis [9]. However, the most specific and potent lymphatic growth factors reported to date are VEGF-C and VEGF-D [10,11], members of the VEGF family. These differ from VEGF-A (also named VEGF) in that they promote proliferation, migration, and survival of lymphatic vascular endothelial cells through the VEGFR-3 signaling pathway [12]. This appears to be a non-redundant function because *VEGF-C*^-/- ^mice are embryonic lethal due to the lack of lymphatic vessels [12]. Under physiologic conditions, VEGF-C is expressed most prominently in the heart, lymph nodes, placenta, and gut [13] but is also expressed by many cancer cells, which can induce lymphatics in metastases. Recent studies reported that VEGF-C is also expressed by CD11b^+ ^myeloid cells that have migrated to inflammatory sites in several animal models of inflammation [14-17], such as corneal transplantation and bacterial lung infection. It was speculated that inflammatory cytokines, such as tumor necrosis factor (TNF) or interleukin 1 (IL-1), stimulate these CD11b^+ ^cells to produce VEGF-C because TNF and IL-1 increase VEGF-C expression in human lung fibroblasts and human umbilical vein endothelial cells *in vitro *[18,19]. However, it has not been formally proven that these cytokines promote VEGF-C expression by CD11b^+ ^cells, and the mechanisms involved are not known.

CD11b antigen is a pan marker for myeloid lineage cells, which also give rise to osteoclasts. We used a combination of fluorescence-activated cell sorting (FACS) and osteoclastogenesis and colony-forming assays to demonstrate that, in peripheral tissues, only CD11b^+^/Gr-1^-/lo ^cells have osteoclastogenic potential [20]. Thus, we termed CD11b^+^/Gr-1^-/lo ^cells osteoclast precursors (OCPs), although this population in bone marrow and spleen also contains macrophage and dendritic cell precursors. Over the last 2 to 3 years, immune cells and molecules that primarily function in immune responses have been demonstrated to affect the functions of cells involved in bone remodeling, particularly osteoclasts [21], which has led to development of the new field of osteoimmunology. We have pursued this line of investigation with OCPs because they belong to a subset of macrophages that can be activated to produce autocrine and paracrine factors that contribute to inflammation and autoimmunity [22]. In our microarray analysis of RNA from OCPs from TNF-transgenic (Tg) and wild-type (WT) mice, we found that VEGF-C expression is significantly increased in cells from TNF-Tg mice, suggesting that VEGF-C-mediated biologic events (for example, lymphangiogenesis) may be involved in the pathogenesis of arthritis. In this study, we used recently developed anti-lymphatic marker antibodies and contrast-enhanced magnetic resonance imaging (MRI) to demonstrate that TNF-Tg mice have a significant increase in the number and size of synovial lymphatic vessels compared with their WT littermates. Furthermore, the severity of erosive synovitis correlates with the lymphatic drainage capacity to local lymph nodes. Together, these findings demonstrate significant changes in lymphatic architecture and draining potential around inflamed joints of arthritic mice and suggest the possible involvement of the lymphatic system in the pathogenesis of inflammatory-erosive arthritis.

## Materials and methods

### Animals

TNF-Tg mice (Tg 3647 line) in a CBA × C57BL/6 background were originally obtained from George Kollias (The Biomedical Sciences Research Center, Greece). We backcrossed them with C57BL6 mice for seven generations [20]. All TNF-Tg mice used in this study were 4 to 8 months old with severe joint synovitis and bone and cartilage destruction. WT littermates were used as controls. TNF-Tg mice were identified by tail polymerase chain reaction (PCR) genotyping and paw deformation. KRN-TCR-Tg mice were obtained from Drs. Diane Mathis and Christophe Benoist (Harvard University, Cambridge, MA, USA) [23]. K/B × N mice were generated by breeding male KRN-TCR-Tg mice with female non-obese diabetic mice (The Jackson Laboratory, Bar Harbor, ME, USA). Serum was collected from 6- to 12-week-old K/B × N arthritic mice, pooled, and stored at -80°C. To generate mice with serum-induced arthritis (SIA), 4- to 5-week-old BALB/c mice were injected with K/B × N serum intraperitoneally (10 μL/gram body weight) on day 1 and day 3. Paw swelling and redness usually occurred the day after the first injection, peaked at 7 to 14 days, and declined thereafter. The Institutional Animal Care and Use Committee of the University of Rochester (Rochester, NY, USA) approved all studies.

### Reagents

Recombinant murine TNF-α was purchased from R&D Systems, Inc. (Minneapolis, MN, USA). Allophycocyanin-, fluorescein isothiocyanate (FITC)-, and phycoerythrin-anti-mouse CD11b (M1/70) were purchased from eBiosciences (San Diego, CA, USA); FITC-anti-mouse Gr-1 (RB6-8C5) from BD Pharmingen (San Diego, CA, USA); rabbit polyclonal antibody to VEGF-C (H-190) from Santa Cruz Biotechnology, Inc. (Santa Cruz, CA, USA); rabbit polyclonal antibody to LYVE-1 from Abcam (Cambridge, MA, USA); mouse monoclonal antibody to CD31 (Mec13.3) from Biocare Medical LLC (Concord, CA, USA); Alexa Fluor 488 goat anti-hamster immunoglobulin G (IgG) from Molecular Probes Inc. (now part of Invitrogen Corporation, Carlsbad, CA, USA); and Alexa Fluor 546 F(ab')_2 _fragment of goat anti-rabbit IgG (H+L) and To-Pro-3 iodid (642/661) were purchased from Invitrogen Corporation.

### Generation of osteoclast precursors

OCPs were generated from two sources:

(a) Spleen. Splenocytes were extracted from spleens of 8- to 12-week-old C57/B6 WT mice through a fine wire mesh, and red blood cells were lyzed with NH_4_Cl (StemCell Technologies, Vancouver, BC, Canada) on ice for 10 minutes. The cells were then washed twice with medium and cultured with conditioned medium from a macrophage colony-stimulating factor (M-CSF)-producing cell line [24] (1:20 dilution) for 3 days to enrich for OCPs, as we described previously [25].

(b) Joints. Ankle and wrist joints were isolated from TNF-Tg mice and WT littermates according to published protocols [26,27] with minor modifications. In brief, mice were sacrificed and skin and muscle were removed from their limbs. Long bones together with a front or rear paw were cut from the limbs. Forceps were used to loosen the joints. The joints were then cut open and digested with 1 mg/mL of collagenase (Sigma-Aldrich, St. Louis, MO, USA) at 37°C for 3 hours with rotation. Cells were then filtered and used for cultures or FACS analysis.

### Affymetrix gene chip analysis

CD11b^+^/Gr-1^-/lo ^OCPs were purified by flow sorting from peripheral blood and bone marrow of TNF-Tg mice and age-matched WT mice by a FACSVantage SE Turbo sorter (Becton, Dickinson and Company, Franklin Lakes, NJ, USA), as we described previously [20,28]. To obtain enough RNA from samples, bone marrow OCPs were pooled from 7 TNF-Tg mice or 11 WT mice and peripheral blood OCPs were from pooled from 7 TNF-Tg mice or 23 WT mice. Two completely independent experiments were performed. Total RNA was prepared using TRIzol (Invitrogen Corporation), processed, and hybridized to MG-U74Av2 gene chips according to Affymetrix protocols (Affymetrix, Santa Clara, CA, USA). Chips were scanned and analyzed using the GeneTraffic (version 3.2) microarray data analysis software (Lobion Informatics, La Jolla, CA, USA). In each group, WT samples were set as the baseline. Data were presented as the fold increase of samples from TNF-Tg mice over WT samples (baseline sample).

### Real-time quantitative reverse transcription-polymerase chain reaction

Total RNA was extracted using TRIzol reagent, and cDNA was synthesized by an RNA PCR Core Kit (Applied Biosystems, Foster City, CA, USA). Quantitative PCR amplification was performed with gene-specific primers using an iCycler iQ Multiple-Color Real-Time PCR Detection System (Bio-Rad Laboratories, Inc., Hercules, CA, USA), as we described previously [25,28]. The primer sequences are listed in Table 1. Expression levels were normalized relative to β-*actin *in the same sample. For each sample, we first obtained the ΔC_T _(difference in threshold cycle values), which is equal to C_T _(target gene) – C_T _(β-actin). We then used control (for example, phosphate-buffered saline [PBS]-treated) values as baseline to obtain the ΔΔC_T_, which is equal to the ΔC_T _of the sample minus the ΔC_T _of the baseline. Finally, we set the baseline as '1', and thus the fold change of each sample is equal to 2^-ΔΔCT^.

**Table 1:** Sequences of primers used in the real-time reverse transcription-polymerase chain reaction

Genes	Sequences of primers	Accession number	Target sites	Size (base pairs)
*VEGF-A*	F: 5'-TTTACTGCTGTACCTCCACCA-3'	M95200	125–423	298
	R: 5'-ATCTCTCCTATGTGCTGGCTTT-3'			
*VEGF-B*	F: 5'-CCTGGAAGAACACAGCCAAT-3'	NM_011697	537–701	165
	R: 5'-GGAGTGGGATGGATGATGTC-3'			
*VEGF-C*	F: 5'-GGGAAGAAGTTCCACCATCA-3'	NM_009506	1258–1392	135
	R: 5'-ATGTGGCCTTTTCCAATACG-3'			
*VEGF-D*	F: 5'-GCTGTCACTGTTGCCCACTA-3'	NM_010216	1423–1611	207
	R: 5'-CCCTTCCTTTCTGAGTGCTG-3'			
*PLGF*	F: 5'-GGGAAGAAGCAAGACATGGA-3'	NM_008827	1055–1261	189
	R: 5'-ATGTCCTGTCCCATCTCCAG-3'			
β-actin	F: 5'-ACCCAGATCATGTTTGAGAC-3'	X03765	280–503	224
	R: 5'-GTCAGGATCTTCATGAGGTAGT-3'			

F, forward primer; PLGF, placental growth factor; R, reverse primer; VEGF, vascular endothelial growth factor.

### Immunofluorescence staining and imaging cytometry

Cells were cytospun on glass slides and fixed by cold methanol at -20°C for 10 minutes. After washing with PBS, cells were incubated in 0.1% Triton and blocked with 1% bovine serum albumin/PBS for 30 minutes at room temperature. The fixed cells were stained with a mixture of FITC-anti-CD11b and anti-VEGF-C antibody followed by Alexa Fluor 546 F(ab')_2 _fragment of goat anti-rabbit IgG (H+L) and by To-Pro-3 iodid.

### Immunohistomorphometry of lymphatic vessels

Joint sections from TNF-Tg and WT mice were fixed in 4.5% phosphate-buffered formalin, decalcified in 14% ethylenediaminetetraacetic acid, and embedded in paraffin wax. Deparaffinized sections were quenched with 3% hydrogen peroxide and treated for antigen retrieval for 30 minutes. Adjacent serial sections were then stained with anti-LYVE-1 or anti-CD31 antibodies. Lymphatic vessels were quantified by a point-counting method, as we described previously [29]. For each mouse, two sections were cut at 250 μm apart and the area and size of LYVE-1^+ ^lymphatic vessels were measured within the synovial tissue. The size and area of lymphatic vessels were expressed per square millimeter of synovium.

### Electrophoretic mobility shift assay

Raw 264.7 osteoclast/macrophage precursor cells were serum-starved overnight and then treated with 10 ng/mL of TNF for 30 and 60 minutes. Nuclear extract preparation and electrophoretic mobility shift assay (EMSA) were performed as described previously [30]. The following double-stranded oligonucleotides were used in this study according to the published mouse VEGF-C promoter sequence [18] (only the top strands are shown): VEGF-C nuclear factor-kappa B (NF-κB)-like sequence (WT), 5'-GCCCAGGGGGGTCCCCGGGAGG-3'; mutated VEGF-C NF-κB-like sequence (mutation underlined), 5'-GCCCAGGGG*AT*TC*T*CCGGGAGG-3'; and SP-1-binding sequence (control; Invitrogen Corporation), 5'-CGAGCCGGCCCCGCCCATC-3'. For competition assays, binding reactions were pre-incubated with unlabeled oligonucleotides for 15 minutes at room temperature.

### *In vivo *contrast-enhanced magnetic resonance imaging

A detailed methodology of this new technique in mice will be published in a separate manuscript [31]. Briefly, mice were positioned with the right leg in a custom-designed murine knee coil and scanned in a Siemens 3 Tesla clinical magnet (Siemens AG, Munich, Germany). A high-resolution fat-suppressed T1-weighted sequence (Sagittal T1-weighted FLASH [fast low-angle shot], repetition time = 45 ms, echo time = 9.03 ms, 192 × 192 pixels, 20 mm × 20 mm field of view, 32 slices of 0.16-mm slice thickness, flip angle = 25°, 1 signal average, time: 8:28) was then initiated. An intravenous injection of Gd-DTPA contrast agent (Omniscan; Amersham Health, now part of GE Healthcare, Little Chalfont, Buckinghamshire, UK) was given, and a post-contrast high-resolution scan (same parameters) was collected. Analysis was performed with Amira 3.1 (TGS; Mercury Computer Systems, Inc., Chelmsford, MA, USA). An image registration and subtraction algorithm on the pre- and post-contrast images in Amira generated an image of the voxels of contrast enhancement. From this image, a three-dimensional (3D) region of interest of muscle tissue was used as a measure of delivered contrast agent, and a threshold of enhancing synovial tissue was generated from this value. Lymph nodes were traced manually and thresholded to define the margin between the node and surrounding fat. Tissue volumes (3D) were reconstructed using a surface generation module in Amira.

### Statistics

Data are presented as mean ± standard deviation of three culture dishes, and all experiments were performed at least twice. Statistical analyses were performed with Statview statistical software (SAS Institute Inc., Cary, NC, USA). Differences among more than two groups were compared using one-way analysis of variance followed by the Mann-Whitney *U *test. *P *values of less than 0.05 were considered statistically significant. Each experiment was repeated at least twice with similar results.

## Results

### VEGF-C expression is upregulated in CD11b^+^/Gr-1^-/lo ^osteoclast precursors from tumor necrosis factor-transgenic mice

**Figure 1:**
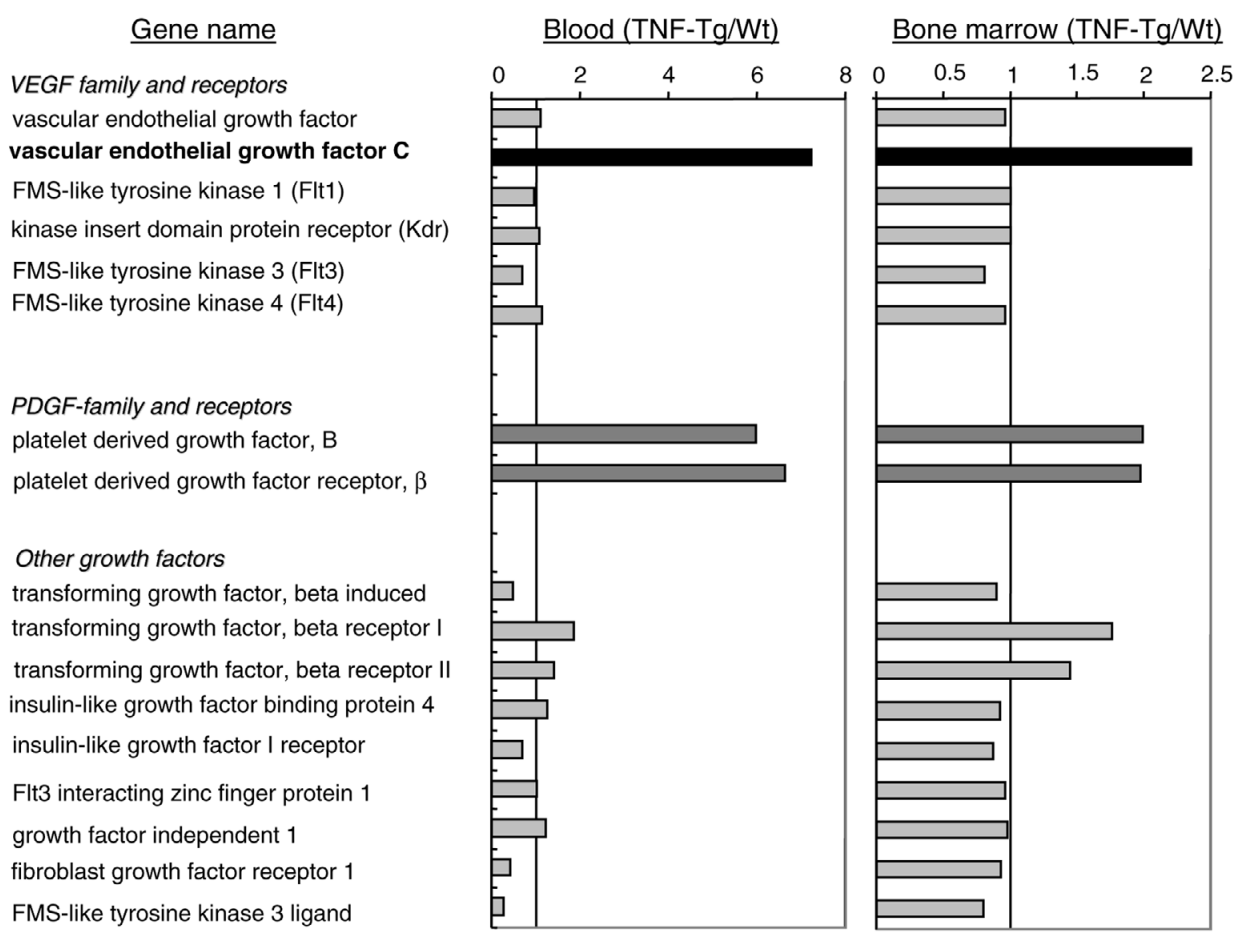
Differential expression of vascular endothelial growth factor (*VEGF*) and platelet-derived growth factor (*PDGF*) family genes in CD11b^+^/Gr-1^-/lo ^osteoclast precursors (OCPs) from tumor necrosis factor-transgenic (TNF-Tg) and wild-type (WT) mice. CD11b^+^/Gr-1^-/lo ^cells from peripheral blood and bone marrow from TNF-Tg (7 mice per array) and WT (23 mice per array) mice were pooled and purified by flow sorting. The RNA samples were subjected to microarray analysis using the GeneChip mouse genome 430A 2.0 array from Affymetrix. The array data on angiogenic gene expression were analyzed using GeneTraffic software. The expression ratio was calculated by dividing the mean value of the intensity of RNA signals from two separate arrays of TNF-Tg OCPs by that from WT cells.

Previous studies demonstrated an increase in circulating OCPs in patients [32] and animals [20] with arthritis and that OCP frequency is reduced in response to anti-TNF therapy, suggesting that OCPs may play important roles in the pathogenesis of arthritis [33]. To screen for novel genes that are differentially expressed by OCPs between arthritic and normal mice, we performed microarrays on RNA isolated from CD11b^+^/Gr-1^-/lo ^OCPs from peripheral blood mononuclear cells and bone marrow pooled from TNF-Tg and WT mice. The purity of CD11b^+^/Gr-1^-/lo ^cells was confirmed by FACS. In our initial bioinformatic screen, we focused on genes encoding angiogenic factors because they are critical for development of inflammation and bone erosion in arthritic joints. Among more than 50 known angiogenic factors (including matrix metalloproteinases, adhesion molecules, enzymes, and growth factors), we found that expression levels of *PDGF-B*, *PDGF receptor β*, and *VEGF-C *were significantly increased in circulating OCPs of TNF-Tg mice (approximately six-fold in TNF-Tg over WT OCPs) (Figure 1). Expression levels of these genes were also increased in bone marrow OCPs but to a lesser extent. Since TNF is known to induce PDGF signaling [34], we decided to explore the possibility that VEGF-C has a novel role in inflammatory arthritis similar to its role in metastatic cancer [35,36].

### Tumor necrosis factor stimulates osteoclast precursors to produce VEGF-C

**Figure 2:**
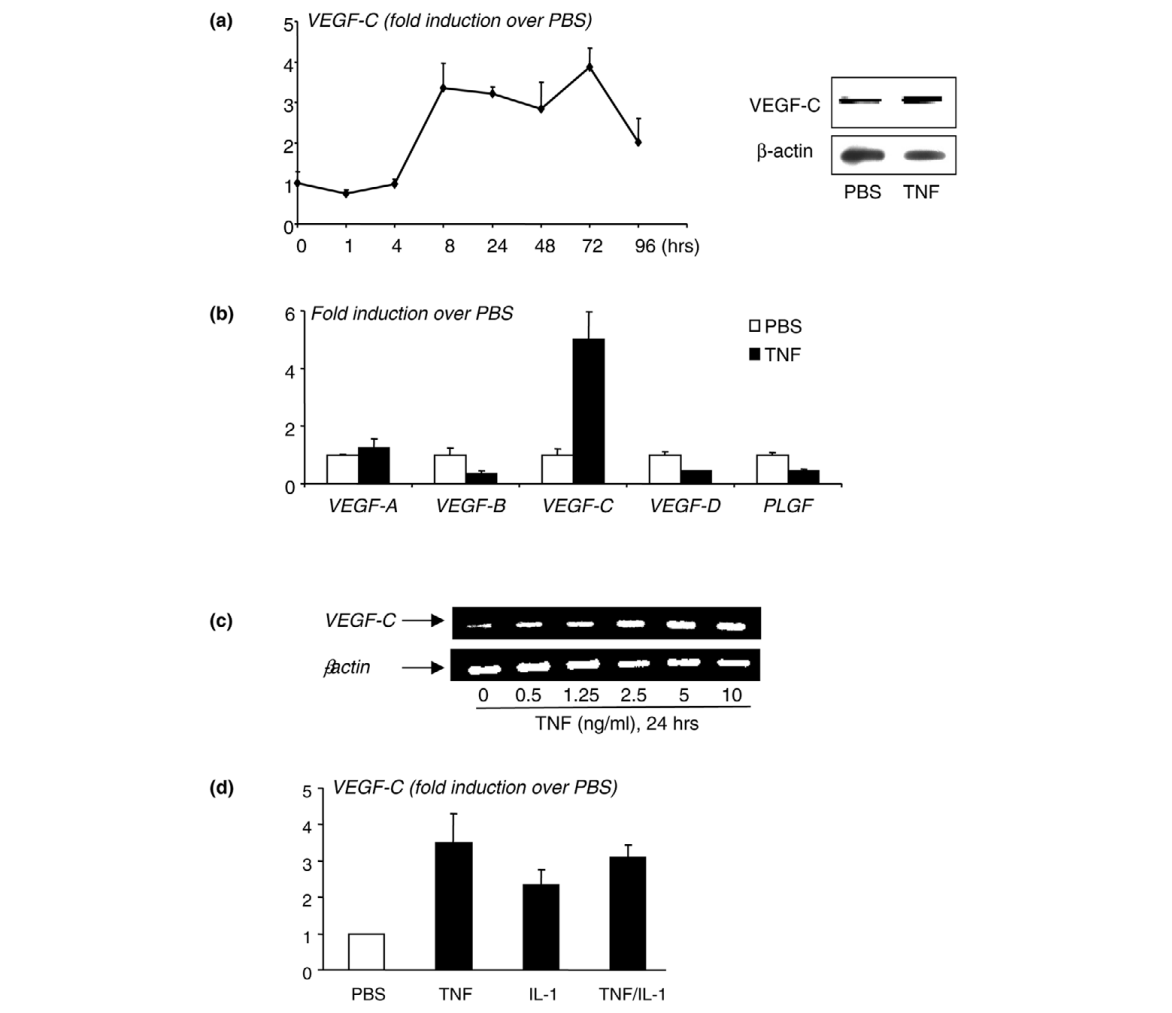
Tumor necrosis factor (TNF) increases expression of vascular endothelial growth factor-C (VEGF-C) by osteoclast precursors (OCPs). Wild-type (WT) spleen cells were cultured with macrophage colony-stimulating factor (M-CSF) for 3 days to enrich for OCPs. OCPs were cultured in 10% serum with M-CSF and treated with phosphate-buffered saline (PBS) or TNF (10 ng/mL). **(a) **Expression of *VEGF-C *and β-actin mRNA was measured by real-time reverse transcription-polymerase chain reaction (RT-PCR) at various times (left panel), and protein levels were measured by Western blot analysis (right panels). **(b) **Expression of *VEGF-A*, -*B*, -*C*, -*D*, and placental growth factor (*PLGF*) mRNA was examined in samples treated for 24 hours. The fold increases in TNF-treated over PBS-treated cells at a given time were calculated. Values are the means of triplicate loadings plus standard deviation. The effect of different doses of TNF **(c) **or a combination with interleukin 1 (IL-1) (TNF and IL-1 10 ng/mL) **(d) **on *VEGF-C *expression at 24 hours was examined by RT-PCR. Experiments were repeated twice with similar results.

To determine whether TNF directly upregulates expression of VEGF-C by OCPs, we cultured WT spleen cells with M-CSF for 3 days to generate OCPs, as we described previously [25]. The rationale for using spleen cells rather than bone marrow cells is that spleen-derived OCPs are closer to circulating OCPs than bone marrow OCPs in terms of their osteoclast-forming potency [37] and the increased *VEGF-C *expression level in circulating OCPs is more than three times higher than in bone marrow OCPs (Figure 1). TNF treatment increased *VEGF-C *mRNA levels by three- to four-fold in a time- and dose-dependent manner, beginning at between 4 and 8 hours, suggesting transcriptional regulation (left panel in Figure 2a). It also increased VEGF-C protein expression at 24 hours (right panels in Figure 2a). TNF did not significantly affect expression of other *VEGF *members (Figure 2b). Dose-response experiments demonstrated that a low dose of TNF (0.5 ng/mL) is sufficient to stimulate VEGF-C protein expression, supporting an *in vivo *role for TNF to induce VEGF-C expression (Figure 2c).

We also examined whether IL-1, another cytokine whose levels are increased significantly in joints of TNF-Tg mice, affects VEGF-C levels in OCPs. Similar to TNF, IL-1 treatment (by 24 hours) also increased *VEGF-C *mRNA by two- to three-fold, but IL-1 + TNF did not have an additive effect (Figure 2d). This suggests that TNF and IL-1 may use a similar signaling pathway to regulate VEGF-C expression.

### Nuclear factor-kappa B mediates tumor necrosis factor-induced VEGF-C expression

**Figure 3:**
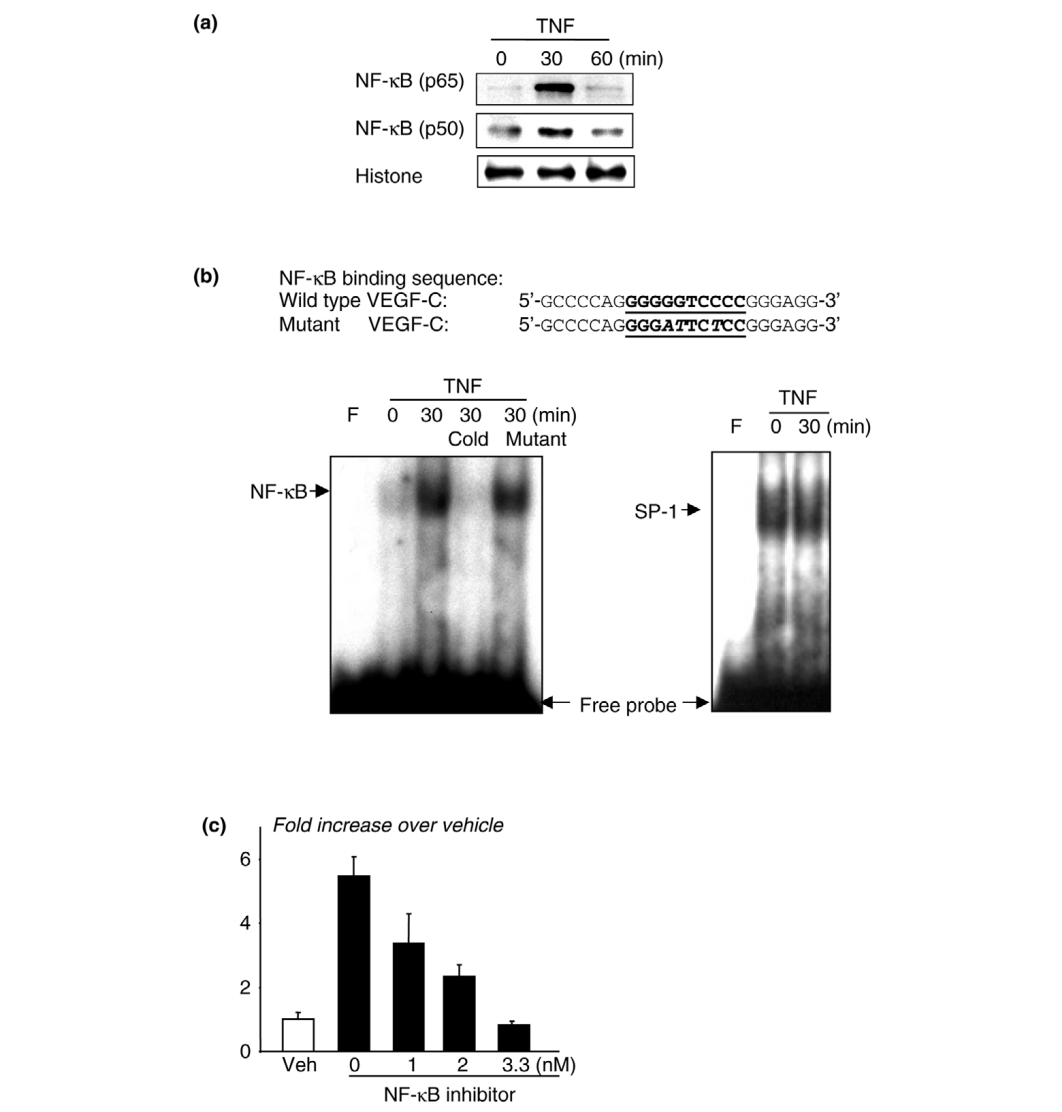
Tumor necrosis factor (TNF) promotes the binding of nuclear proteins to the nuclear factor-kappa B (NF-κB) binding sequences of the vascular endothelial growth factor-C (VEGF-C) promoter. Raw264.7 osteoclast/macrophage precursors were cultured in 0.5% bovine serum albumin overnight. Cells were treated with TNF for 30 to 60 minutes. Nuclear extracts were isolated and subjected to Western blot analysis using anti-NF-κB p65 and p50 antibodies **(a) **or to electrophoretic mobility shift assay using a ^32^P-labeled probe consisting of the putative NF-κB binding sequence of the mouse VEGF-C promoter **(b)**. The specificity of binding was confirmed by using 50-fold more unlabeled wild-type (WT) or mutated probe in which the putative NF-κB binding sequence was mutated and could not be bound by NF-κB in a competition reaction. An SP-1 probe was used as a loading control. WT osteoclast precursors were treated with TNF ± an NF-κB inhibitor for 24 hours, and expression of *VEGF-C *was determined by real-time reverse transcription-polymerase chain reaction. Values are the means of three mice plus standard deviation. **(c) **The fold decrease in expression in the NF-κB inhibitor-treated over vehicle-treated cells was calculated. Experiments were repeated two times with similar results.

NF-κB is a transcription factor that mediates induction of genes by TNF in many cell types, including OCPs [38]. NF-κB also mediates heregulin-beta-1-induced VEGF-C expression in human breast cancer cells [39]. We found that there is a putative NF-κB-binding element, GGGGTCCC, at the -108/-99 region of the mouse *VEGF-C *promoter, which is at a location similar to that in the human promoter [18]. Thus, to determine whether TNF stimulates binding of NF-κB proteins to this element in OCPs, we treated Raw 264.7 cells with TNF for 30 and 60 minutes and performed Western blot analysis and EMSA on nuclear extracts from the cells. TNF treatment for 30 minutes increased nuclear translocation of NF-κB p65 and p50 proteins (Figure 3a) and specific binding of NF-κB to the VEGF-C DNA probe (Figure 3b). To further confirm the involvement of NF-κB in TNF-induced VEGF-C expression, we treated WT OCPs with an NF-κB inhibitor (Calbiochem, now part of EMD Biosciences, Inc., San Diego, CA, USA) and found that it inhibited TNF-induced VEGF-C expression in a dose-dependent manner (Figure 3c). These data support TNF induction of VEGF-C expression by activation of NF-κB.

### CD11b^+^/Gr-1^-/lo ^cells isolated from joints of tumor necrosis factor-transgenic mice express high levels of VEGF-C

**Figure 4:**
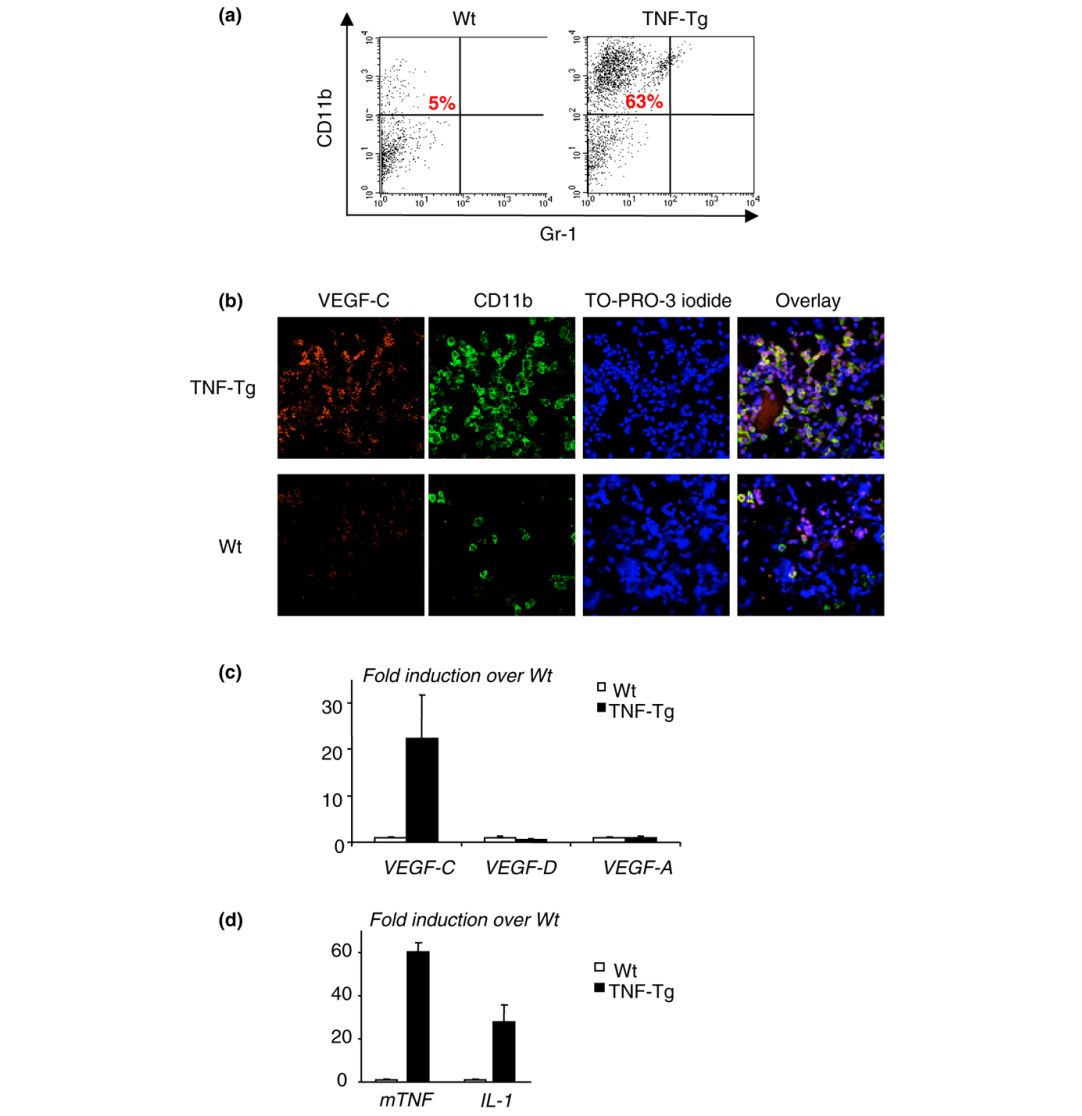
Cells from joints of tumor necrosis factor-transgenic (TNF-Tg) mice express high levels of vascular endothelial growth factor-C (VEGF-C). Ankle and wrist joints of TNF-Tg mice and wild-type (WT) littermates were subjected to collagenase digestion to isolate cells, which then were stained with fluorescein isothiocyanate (FITC)-anti-CD11b and phycoerythrin-anti-Gr-1 and subjected to fluorescence-activated cell sorting analysis. **(a) **The percentage of CD11b^+^/Gr-1^-/lo ^cells is shown in a representative histogram from one pair of TNF-Tg and WT mice. Cells (3 × 10^5^) were cytospun onto glass slides and double-stained with FITC-anti-CD11b and rabbit anti-VEGF-C followed by anti-rabbit Alexa 546. TO-PRO-3 iodide was used as a DNA dye for nuclear staining. **(b) **Co-localization of CD11b and VEGF-C proteins was observed, and pictures were taken under a confocal microscope at a power of × 20. Cells were cultured with M-CSF for 3 days, and adherent cells were harvested. **(c) **Expressions of *VEGF-A*, -*C*, and -*D *mRNA were measured by real-time reverse transcription-polymerase chain reaction (RT-PCR). The fold increases in expression in TNF-Tg over WT cells were calculated. Values are the means of triplicate loadings plus standard deviation (SD). **(d) **The expression levels of TNF and IL-1 mRNA in joint extracts from TNF-Tg mice and WT littermates were determined by real-time RT-PCR. Values are the means of three mice plus SD. Experiments were repeated using four additional pairs of TNF-Tg and WT mice with similar results.

To examine whether OCPs at the site of inflammation express VEGF-C, we isolated cells from joints of TNF-Tg mice and WT littermates using a sequence enzyme digestion method [26,27]. We obtained, on average, a total of 1.5 × 10^6 ^cells from the four paws of a TNF-Tg mouse. FACS analysis revealed that approximately 60% of joint cells from TNF-Tg mice are CD11b^+ ^and almost all of them are Gr-1^- ^(Figure 4a). Thus, in subsequent experiments, we used only CD11b as a single marker for OCPs. To determine whether CD11b^+ ^cells express VEGF-C, we performed double immunofluorescence staining using anti-CD11b and VEGF-C antibodies in a cytospin preparation of these cells. There were much more CD11b and VEGF-C double-stained cells in the joints of TNF-Tg mice than in WT mice (Figure 4b). We also found that most of the VEGF-C^+ ^cells are CD11b^+^, whereas no CD11b^- ^cells are VEGF-C^-^, suggesting that VEGF-C is produced in arthritic joints by OCPs. To examine whether these joint CD11b^+ ^cells specifically produce more VEGF-C than other VEGFs, we cultured them with M-CSF for 3 days, harvested adherent cells, and performed real-time reverse transcription-PCR to assess the expression level of *VEGF-A*, *VEGF-C*, and *VEGF-D*. The rationale for using these M-CSF-dependent adherent cells is that they are composed mainly of OCPs and monocytes, as we described previously [25]. We found that M-CSF-dependent cells that were derived from joints of TNF-Tg mice expressed much higher levels of *VEGF-C *compared with those of WT mice (Figure 4c) whereas *VEGF-A *and *VEGF-D *levels were similar, suggesting that OCPs/monocytes are the major source of VEGF-C in arthritic joints of TNF-Tg mice. To confirm that there is elevated TNF or IL-1 expression in the joints of TNF-Tg mice, we measured the expression levels of *TNF *and *IL-1 *mRNA and demonstrated that they were remarkably increased compared with levels in WT joints (Figure 4d), which supports the hypothesis that local increased cytokines stimulate VEGF-C production.

### Increased lymphangiogenesis in joints of tumor necrosis factor-transgenic mice

**Figure 5:**
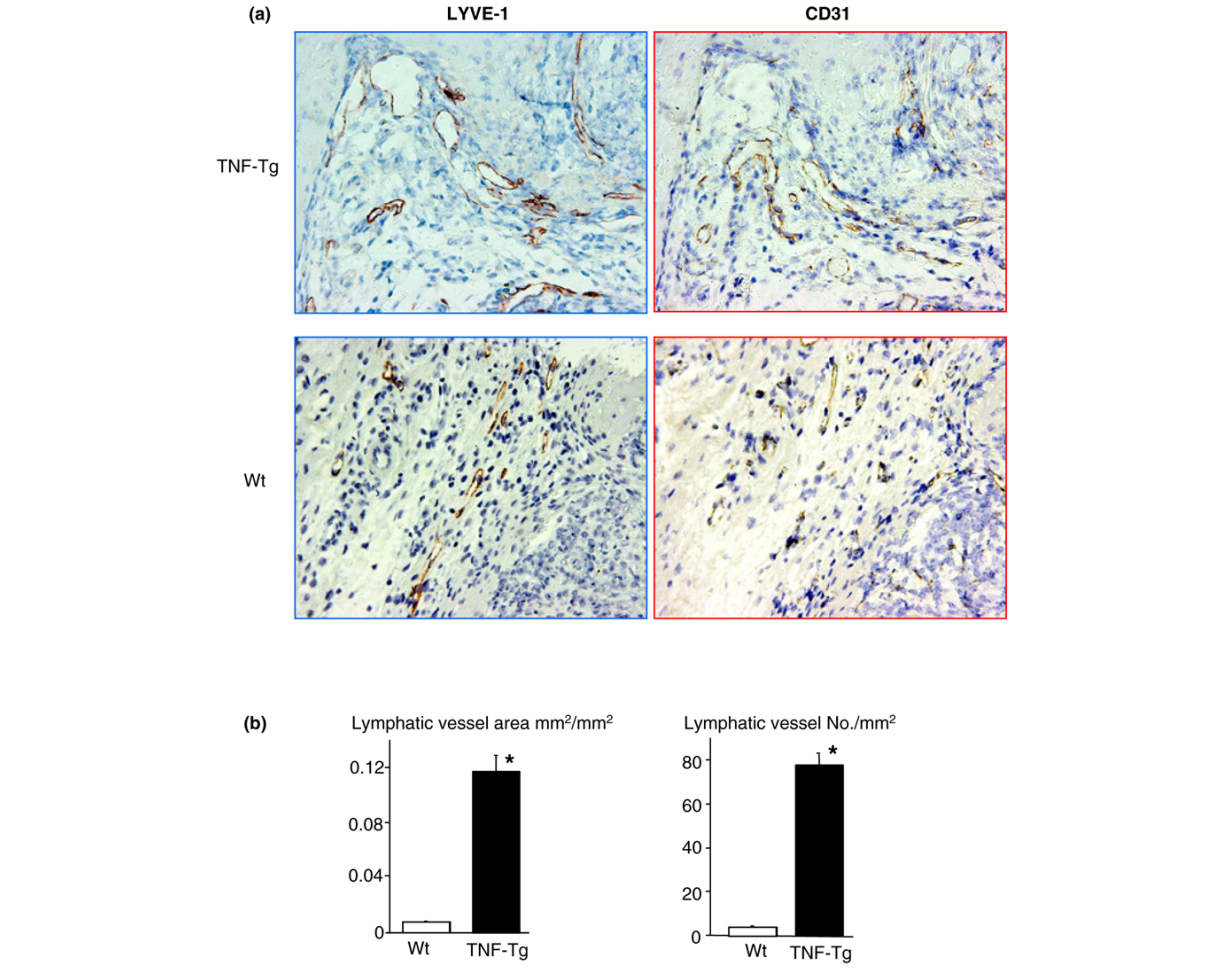
Enlargement of lymphatic vessels in the joints of tumor necrosis factor-transgenic (TNF-Tg) mice. Joint sections from TNF-Tg mice or wild-type (WT) littermates were immunostained with anti-LYVE-1 or CD31 antibody. **(a) **Micrographs (× 20) show increased numbers and diameters of LYVE-1^+ ^lymphatic vessels in the synovium of TNF-Tg mice. **(b) **The area and number of lymphatic vessels within the synovium were counted. Values are the means plus standard deviation of five TNF-Tg mice and six WT mice. *p < 0.05 versus WT samples. LYVE-1, lymphatic endothelial hyaluronan receptor 1.

**Figure 6:**
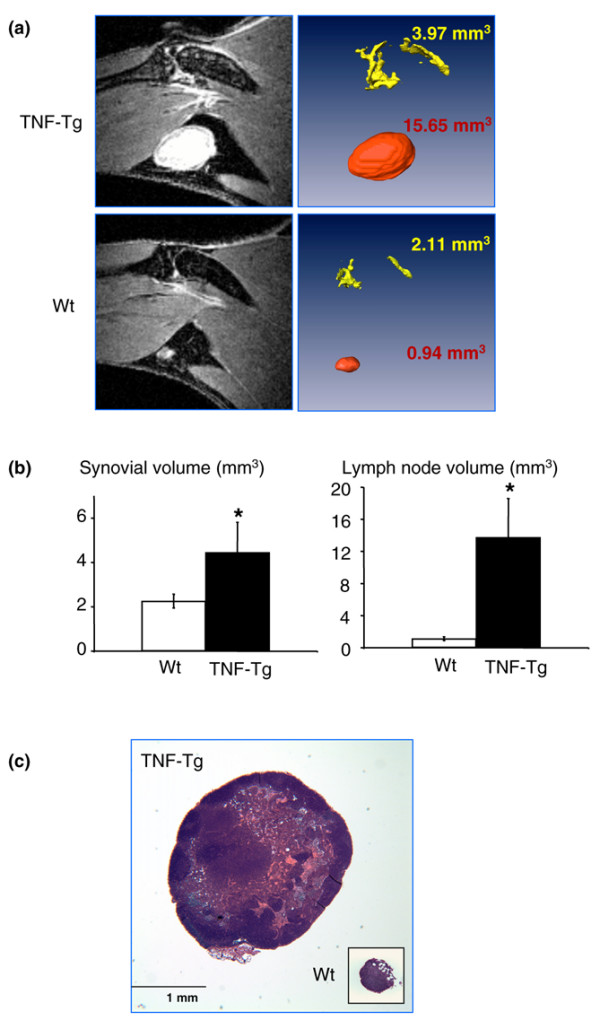
Increased volume of popliteal lymph nodes in tumor necrosis factor-transgenic (TNF-Tg) mice. **(a) **A representative post-contrast magnetic resonance imaging slice from a TNF-Tg and wild-type (WT) mouse (5 months old). **(b) **Using a semi-automated segmentation procedure in Amira software, the synovial (yellow) and lymph node (red) volumes were quantified and visualized in TNF-Tg and WT mice. *p < 0.05 versus WT samples (*n *= 5). **(c) **Representative hematoxylin-and-eosin sections (× 4) demonstrate differences in lymph node size between TNF-Tg and WT animals.

Since VEGF-C is a specific lymphatic growth factor [40] and VEGF-C expression is increased in OCPs, we sought to determine whether there was increased lymphangiogenesis in joint sections from TNF-Tg mice compared with WT mice using immunohistochemistry and an antibody to the lymphatic endothelial cell marker, LYVE-1. We also assessed, as a positive control, blood vessels lined by endothelial cells using a CD31 antibody because these are known to be increased in TNF-Tg versus WT joints [41]. These results clearly demonstrated large LYVE-1^+ ^lymphatic vessels compared with smaller CD31^+ ^vessels in the pannus of TNF-Tg mice (Figure 5a). Histomorphometric quantitation of the area and number of these lymphatic vessels confirmed a significant increase in TNF-Tg versus WT joints (Figure 5b). To assess the functional significance of this increased lymphangiogenesis, we used a new *in vivo *MRI technology that we have developed [31] to see whether the draining popliteal lymph nodes from the knee and ankle joints of TNF-Tg mice with synovitis are increased versus WT littermates. Figure 6a demonstrates the remarkable differences in 2D MRI and 3D reconstructed images of the synovium and popliteal lymph nodes of TNF-Tg versus WT mice. Volumetric analyses confirmed that TNF-Tg mice have significantly larger synovial and popliteal lymph node volumes compared with WT mice (Figure 6b) and these were confirmed with histology (Figure 6c).

**Figure 7:**
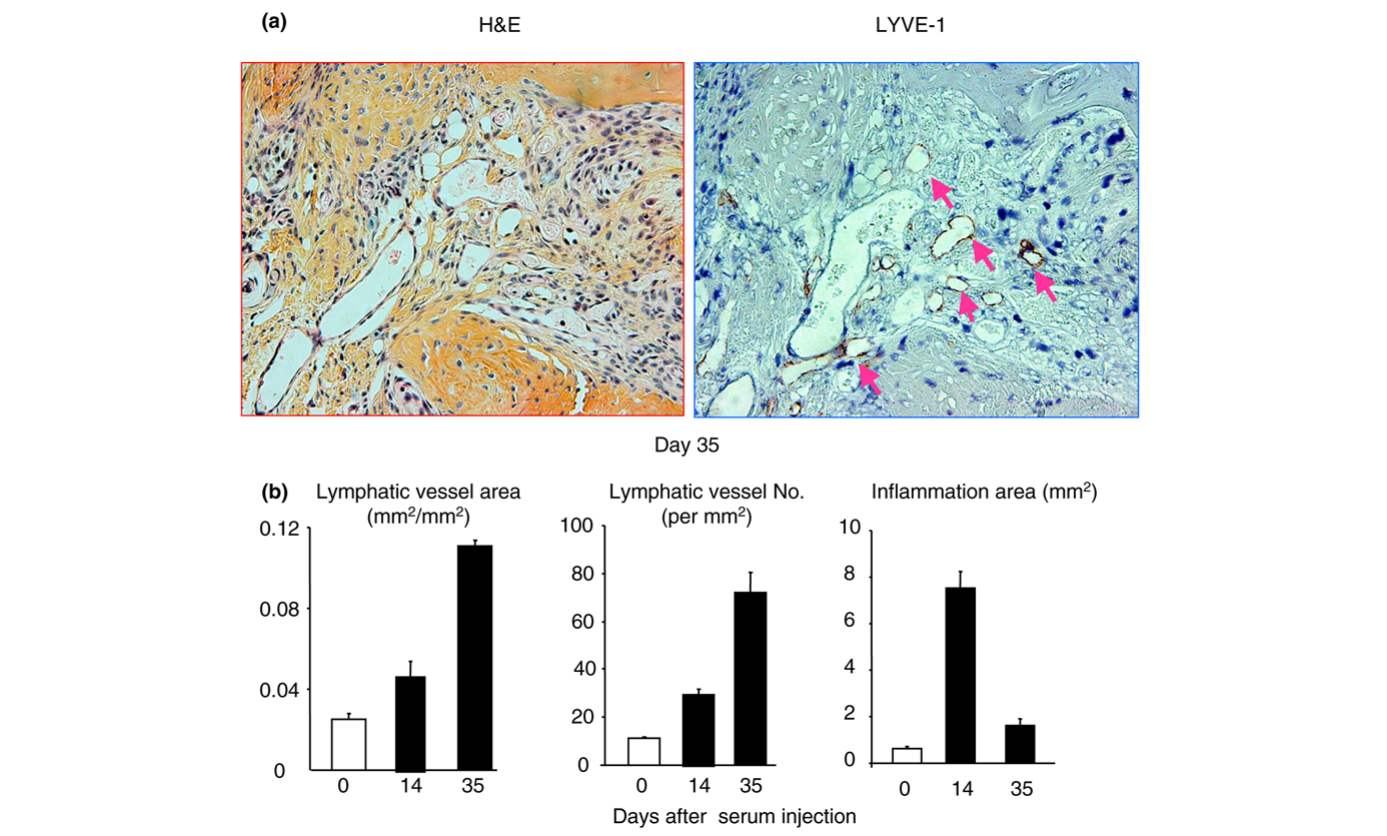
Increased lymphangiogenesis in joints of mice with serum-induced arthritis. Wild-type mice received serum from K/B × N mice to induce arthritis and were sacrificed at 0, 14, and 35 days after serum injection (*n *= 3 or 4 mice at each time point). Ankle joint sections were immunostained with anti-LYVE-1 antibody. **(a) **Representative pictures show inflamed pannus and large numbers of LYVE-1^+ ^lymphatic vessels in an adjacent section (pink arrows) at day 35. **(b) **The area and number of lymphatic vessels within the pannus were determined by histomorphometric analysis. Values are the means plus standard deviation of 3 or 4 mice at each time point. H&E, hematoxylin and eosin; LYVE-1, lymphatic endothelial hyaluronan receptor 1.

To demonstrate that increased lymphangiogenesis is not limited to the arthritis in TNF-Tg mice and to determine the association between joint inflammation and lymphatic vessel formation during the course of arthritis induction, we used mice with K/B × N SIA. This SIA model has clearly distinguishable phases of disease progression and uniform severity of joint lesions among animals compared with TNF-Tg mice. The SIA mice were sacrificed at 0, 14, and 35 days after K/B × N serum injection, and joint samples were subjected to histology and LYVE-1 immunostaining. At day 14 after serum injection, mice developed severe joint inflammation with pannus formation and inflammatory cell infiltration, which is accompanied with increased lymphatic vessel area and number (Figure 7). By day 35, the inflammation declined, but the lymphatic vessel formation had increased further (Figure 7) to a degree similar to that seen in TNF-Tg mice (Figure 5). These data suggest that increased lymphangiogenesis is a common phenotype of inflammatory-erosive arthritis.

## Discussion

Patients with RA often accompany with enlarged draining lymph nodes [42] and increased lymph flow rates [43] in affected limbs, which are correlated with inflamed synovial volume. However, a cellular and molecular mechanism to explain these changes has yet to be postulated. In the present study, we demonstrated that CD11b^+^/Gr-1^-/lo ^OCPs from joints of TNF-Tg mice produce high levels of the lymphatic growth factor, VEGF-C, and that joints from mice in two models of RA have increased numbers of lymphatic vessels and enlargement of draining popliteal lymph nodes. Thus, lymphangiogenesis is also significantly increased in joints of mice with inflammatory arthritis. To date, most studies of inflammation-induced lymphangiogenesis have been performed in animal models in which the examined tissues have included cornea, lung, and skin [15,17,44], because frozen sections from these tissues can be prepared readily for immunostaining to identify lymphatic vessels and because dye injection can be used to examine the lymphatic drainage. However, it is difficult to apply these methods to joints. Here, we used a combination of microarrays, FACS, immunohistochemistry, and a novel *in vivo *contrast-enhanced-MRI technique to demonstrate that the lymphatic vasculature in inflamed joints and draining lymph nodes are significantly increased in mice with TNF and SIA. Our findings are consistent with those in clinical studies demonstrating increased VEGF-C expression in RA joints [45,46] and increased size of lymph nodes [43]. Furthermore, based on increased volume of collected lymph [42] and our demonstration of increased VEGF-C production by joint OCPs, we propose that the development of large lymphatic vessels in the pannus results from proliferation of lymphatic endothelial cells and their distention by increased amounts of lymph.

While the origin of lymphatic endothelia cells remains an area of active research, several studies on inflamed corneas, skin, and lung have reported the presence of CD11b^+ ^myeloid cells expressing VEGF-C in these tissues [15,47]. These studies speculated that inflammatory cytokines stimulate VEGF-C production by these CD11b^+ ^cells based on previous work in human lung fibroblasts and human umbilical vein endothelial cells [18,19]. Here, we demonstrated that TNF and IL-1 upregulate VEGF-C expression in CD11b^+ ^OCPs. To validate our microarray findings and provide insight into the mechanism of TNF-induced VEGF-C expression in OCPs, we provide preliminary evidence indicating that this response is mediated by NF-κB-dependent transcription. Since other signal transduction pathways could also be involved and TNF could be acting indirectly through prostaglandins [48], which also mediate *VEGF-C *transcription in cancer cells [49], future studies are needed to elucidate the mechanisms of inflammation-induced VEGF-C expression in OCPs.

Considering the cellular heterogeneity of joint pannus, it is important to determine the primary source of VEGF-C in arthritic joints. While our studies focused on OCPs, others have shown that TNF and/or IL-1 stimulates VEGF-C expression by human lung fibroblasts, blood vascular endothelial cells, and synovial cells [19,46]. We found that TNF also stimulates VEGF-C expression in NIH3T3 and C2C12 fibroblast cell lines (data not shown), suggesting that fibroblast-like cells in synovium could perhaps be another source of VEGF-C in arthritic pannus. However, these results were obtained from *in vitro *treatment of cells with cytokines and may not precisely reflect the *in vivo *situation, particularly in the joint local microenvironment. Our immunocytochemistry studies using cells freshly isolated from joints demonstrated that most of the VEGF-C-expressing cells are CD11b^+ ^(Figure 4b). One potential concern with this approach is that primary joint cells are composed of a mixture of cell types. To address this limitation, we cultured these cells with M-CSF and used only adherent cells for further study. Under these culture conditions, more than 90% of adherent cells are CD11b^+^/Gr-1^-/lo ^OCPs (Q. Zhang, Y. Lu, S. Proulx, R. Guo, Z. Yao, E.M. Schwarz, B.F. Boyce, L. Xing, unpublished data). We found that these M-CSF-dependent joint cells express much higher levels of VEGF-C than cells treated *in vitro *(20- to 30-fold increase in joint cells in Figure 3c versus 3- to 5-fold increase in TNF-treated cells in Figure 2a). Thus, although other cell types may also be VEGF-C-producing cells, CD11b^+^/Gr-1^-/lo ^OCPs likely are one of the major sources of VEGF-C in joint pannus.

Our findings demonstrated that increased lymphangiogenesis is associated with the progression of joint inflammation, which occurs not only in dysregulated TNF-induced arthritis (Figure 5) but also in mice with SIA (Figure 7). A recent study reported that, in joint sections of collagen-induced arthritis, the number of LYVE-1^+ ^lymphatic vessels is increased [50]. Thus, elevated lymphangiogenesis likely is a common feature of inflammatory arthritis. Inflammation-induced lymphangiogenesis in joints appears to be a slow process, taking 2 to 3 weeks (Figure 7). Our explanation is that OCPs or other VEGF-C-producing cells may need to migrate to the inflamed joints first and then respond to elevated cytokine levels to produce VEGF-C, which then stimulates formation of lymphatic endothelial cells.

Interestingly, increased lymphatic vasculature persists even after serum-induced inflammation has resolved (Figure 7). This is consistent with our observation of no change in lymphatic vessel area or number in TNF-Tg mice with a significant reduction in their joint inflammation after anti-TNF therapy treatment (see Additional File 1). Persistent lymphangiogenesis was also reported in a mouse model of chronic respiratory tract infection in which inflammation has been cured by antibiotics [17]. Currently, there is no explanation why these lymphatic vessels do not disappear along with the reduction in inflammation. One speculation is that lymphatic enlargement makes affected tissues more susceptible to later inflammation by facilitating the accumulation of immune cells at the site of injury or infection [51]. However, it is also possible that an increased lymphatic network will prime tissues to respond to acute inflammation.

A central question that arises from our study and other recent studies regarding inflammation-induced lymphangiogenesis is whether lymphangiogenesis in arthritis is beneficial or harmful to affected joints. Early clinical studies proposed that inflammation-driven lymphangiogenesis induces the expansion of the lymphatic network in an exacerbated manner such that the lymphatic vessels may be dysfunctional, as reported in psoriasis and Crohn disease [52-54]. Recently, VEGF-C or VEGFR-3 antagonists have been used to directly stimulate or block VEGF-C/VEGFR-3-mediated lymphangiogenesis in several models of inflammation. In general, the effects of manipulating lymphangiogenesis in inflammatory conditions are not clear and appear to be largely dependent on the type of model used. For example, in the corneal transplantation model, lymphangiogenesis and angiogenesis are increased by grafting, and blockade of either form of new vessel formation has a similar beneficial effect and prevents graft rejection [55]. In contrast, UVB-induced skin inflammation is exacerbated by VEGF-C antagonism [56], suggesting that stimulation of the lymphatic system might reduce disease severity. Thus, the functional importance of lymphangiogenesis in the pathogenesis of RA remains to be determined.

## Conclusion

Our findings demonstrate that lymphangiogenesis is significantly increased in joints of mice with inflammatory arthritis. TNF and OCPs appear to play a critical role in initiating this process through stimulation of VEGF-C production.

## Abbreviations

3D = three-dimensional; C_T _= threshold cycle; EMSA = electrophoretic mobility shift assay; FACS = fluorescence-activated cell sorting; FITC = fluorescein isothiocyanate; IgG = immunoglobulin G; IL-1 = interleukin 1; LYVE-1 = lymphatic endothelial hyaluronan receptor 1; M-CSF = macrophage colony-stimulating factor; MRI = magnetic resonance imaging; NF-κB = nuclear factor-kappa B; OCP = osteoclast precursor; PBS = phosphate-buffered saline; PCR = polymerase chain reaction; PDGF = platelet-derived growth factor; RA = rheumatoid arthritis; SIA = serum-induced arthritis; Tg = transgenic; TNF = tumor necrosis factor; VEGF = vascular endothelial growth factor; VEGFR-3 = vascular endothelial growth factor receptor 3; WT = wild-type.

## Competing interests

The authors declare that they have no competing interests.

## Authors' contributions

LX participated in study design; acquisition, analysis, and interpretation of data; and manuscript preparation and had full access to all of the data in the study and takes responsibility for the integrity of the data and the accuracy of the data analysis. QZ and STP participated in study design; acquisition, analysis, and interpretation of data; manuscript preparation; and statistical analysis. YL participated in study design; acquisition, analysis, and interpretation of data; and statistical analysis. EMS and BFB participated in study design, analysis and interpretation of data, and manuscript preparation. RG and ZY participated in acquisition, analysis, and interpretation of data. All authors read and approved the final manuscript.

## Supplementary Material

Additional file 1 Persistence of lymphatic vasculature in joints of tumor necrosis factor-transgenic (TNF-Tg) mice that received anti-TNF therapy. TNF-Tg mice (2.5 months old) received placebo or anti-TNF antibody (10 mg/kg per week × 8 weeks). Ankle sections were immunostained with anti-LYVE-1 antibody. The area and number of LYVE-1^+ ^lymphatic vessels within the pannus and the area of inflammation per ankle were assessed. Values are the means plus standard deviation of three placebo- or anti-TNF-treated mice. No statistically significant difference was observed between values in the placebo- and anti-TNF-treated group. TNF antibody treatment significantly reduced the inflammation. *p < 0.05 anti-TNF-treated group compared with placebo group. LYVE-1, lymphatic endothelial hyaluronan receptor 1. Click here for file
