# Supplementary material for: Increased lymphangiogenesis in joints of mice with inflammatory arthritis
Source: Arthritis Res Ther. 2007 Nov 12;9(6):R118. doi: 10.1186/ar2326 (PMC2246237; doi:10.1186/ar2326)
Supplement: Additional file 1 — Persistence of lymphatic vasculature in joints of tumor necrosis factor-transgenic (TNF-Tg) mice that received anti-TNF therapy. TNF-Tg mice (2.5 months old) received placebo or anti-TNF antibody (10 mg/kg per week × 8 weeks). Ankle sections were immunostained with anti-LYVE-1 antibody. The area and number of LYVE-1+ lymphatic vessels within the pannus and the area of inflammation per ankle were assessed. Values are the means plus standard deviation of three placebo- or anti-TNF-treated mice. No statistically significant difference was observed between values in the placebo- and anti-TNF-treated group. TNF antibody treatment significantly reduced the inflammation. *p < 0.05 anti-TNF-treated group compared with placebo group. LYVE-1, lymphatic endothelial hyaluronan receptor 1. [file ar2326-S1.ppt]

## Slide 1
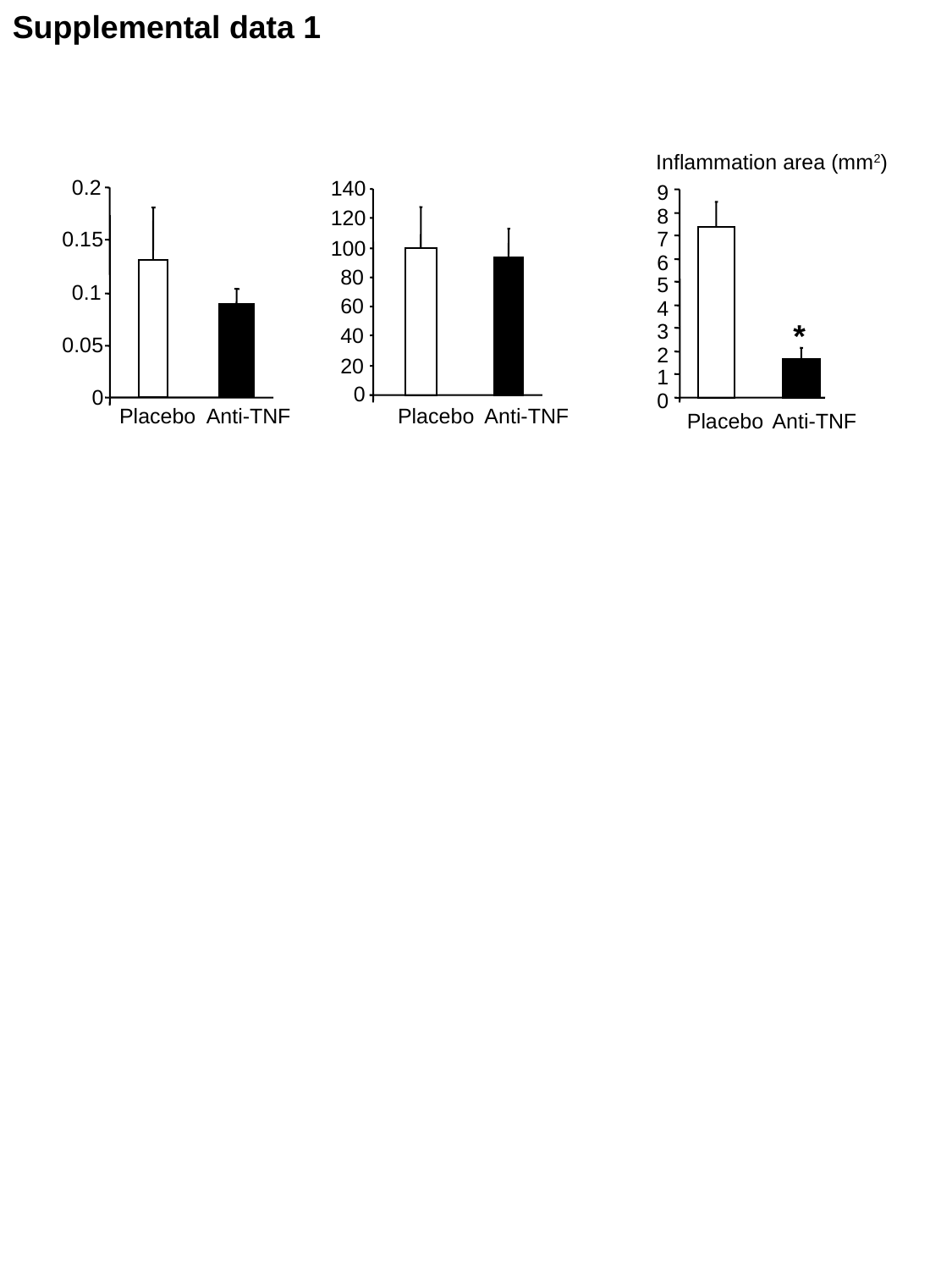

Supplemental data 1
Lymphatic vessel area
mm2/mm2
Lymphatic vessel #/mm2
0.2
140
120
0.15
100
80
0.1
60
40
0.05
20
0
0
Placebo
Anti-TNF
Placebo
Anti-TNF
Inflammation area (mm2)
9
8
7
6
5
4
3
2
1
0
Placebo
Anti-TNF
*
